# An Atlas of Plant Transposable Elements

**DOI:** 10.12688/f1000research.74524.1

**Published:** 2021-11-24

**Authors:** Daniel Longhi Fernandes Pedro, Tharcisio Soares Amorim, Alessandro Varani, Romain Guyot, Douglas Silva Domingues, Alexandre Rossi Paschoal

**Affiliations:** 1 Department of Computer Science; Bioinformatics and Pattern Recognition Group, Graduation Program in Bioinformatics, Federal University of Technology - Paraná (UTFPR), Cornélio Procópio, Paraná, 86300000, Brazil; 2 Departament of Agricultural and Environmental Biotechnology, School of Agricultural and Veterinary Sciences, São Paulo State University (UNESP), Jaboticabal, São Paulo, 14884-900, Brazil; 3 Institut de Recherche pour le Développement, IRD, University of Montpellier, Montpellier, France; 4 Department of Electronics and Automatization, Universidad Autónoma de Manizales, Manizales, Colombia; 5 Group of Genomics and Transcriptomes in Plants, Institute of Biosciences, São Paulo State University (UNESP), Rio Claro, São Paulo, 13506-900, Brazil

**Keywords:** mobile elements, atlas, large-scale, genome-wide, standardized, plants

## Abstract

Advances in genomic sequencing have recently offered vast opportunities for biological exploration, unraveling the evolution and improving our understanding of Earth biodiversity. Due to distinct plant species characteristics in terms of genome size, ploidy and heterozygosity, transposable elements (TEs) are common characteristics of many genomes. TEs are ubiquitous and dispersed repetitive DNA sequences that frequently impact the evolution and composition of the genome, mainly due to their redundancy and rearrangements. For this study, we provided an atlas of TE data by employing an easy-to-use portal ( APTE website ). To our knowledge, this is the most extensive and standardized analysis of TEs in plant genomes. We evaluated 67 plant genomes assembled at chromosome scale, recovering a total of 49,802,023 TE records, representing a total of 47,992,091,043 (~47,62%) base pairs (bp) of the total genomic space. We observed that new types of TEs were identified and annotated compared to other data repositories. By establishing a standardized catalog of TE annotation on 67 genomes, new hypotheses, exploration of TE data and their influences on the genomes may allow a better understanding of their function and processes. All original code and an example of how we developed the TE annotation strategy is available on GitHub ( *Extended data*).

## Introduction

The growing number of sequenced plant genomes is providing unprecedented opportunities for biological studies, evolution, and growing of many algal and Viridiplantae species. We estimate more than 13k plant genomes have been released (NCBI), revealing that plant genomes are faintly explored. High diversity in terms of ploidy, heterozygosity, and genome size, probably due to a dynamic set of old and recent bursts of transposable elements (TEs), are common hallmarks of many plant genomes. ^1^ TEs can comprise between 32% to 56% ( *Utricularia* genomes), ^2^^,^^3^ to up to 90% in many plant genomes, ^4^^–^^6^ e.g., maize ^7^ and wheat. ^5^^,^^8^^,^^9^

TEs can be organized into two main classes. Each class is hierarchically organized into orders, superfamilies, families, and subfamilies. This terminology is primarily associated with the type of their transposition mechanisms. ^3^^,^^10^ They are classified into: (i) retrotransposons (Class I), which are propagated by a “copy-and-paste” mobility mechanism and are the most redundant TE class in plant genomes; and (ii) DNA transposons (Class II), which are known for the “cut-and-paste” mechanism that allows them to move to a completely different position. Moreover, both Classes may contain autonomous members ^10^^,^^11^ for which the transposition mechanism depends on an autonomous and cognate type of TE. ^11^^,^^12^

However, despite their significance, in-depth identification and analysis of TEs content in the sequenced plant genomes remains barely explored. ^13^^,^^14^ The lack of concise data available may prevent the enrichment of *in silico*, functional genomics research and compromises the appearance of new strategies to investigate TEs. Recently, many computational models and entire wet-lab efforts have increasingly been helping to understand these sequences. ^15^^–^^18^ For example, Ensembl Plants ^19^ provides high-quality, primary genomic information for 67 plant (in the broad sense, including green plants, green and red algae) genomes, assembled near or at chromosome scale; however, mobile sequences are poorly systematized and have a humble coverage.

These observations prompted us to standardize tools and methods aiming to improve TE detection, annotation and standardization. In this work, we developed a new method for systematic annotation of plant TEs, using the 67 genomes available at Ensembl Plants assembled at chromosome scale as a starting point. Our identification was standardized, applying the same methodologies to all genomes and delivering a concise Atlas of TEs annotation in plant genomes. We also provided an updated analysis of non-coding RNAs (ncRNAs) overlapping TEs. This annotation is accessible on the Atlas website for exploration and download, which might be relevant to any type of research involving mobile sequences.

## Methods

### Data source

All genomes (Supplementary Material 1, *Extended data*) were downloaded from the Ensembl Plants ^19^ database, version 41 (57 genomes) and 45 (plus 10 new genomes).

### Annotation of transposable elements

**Figure 1.**
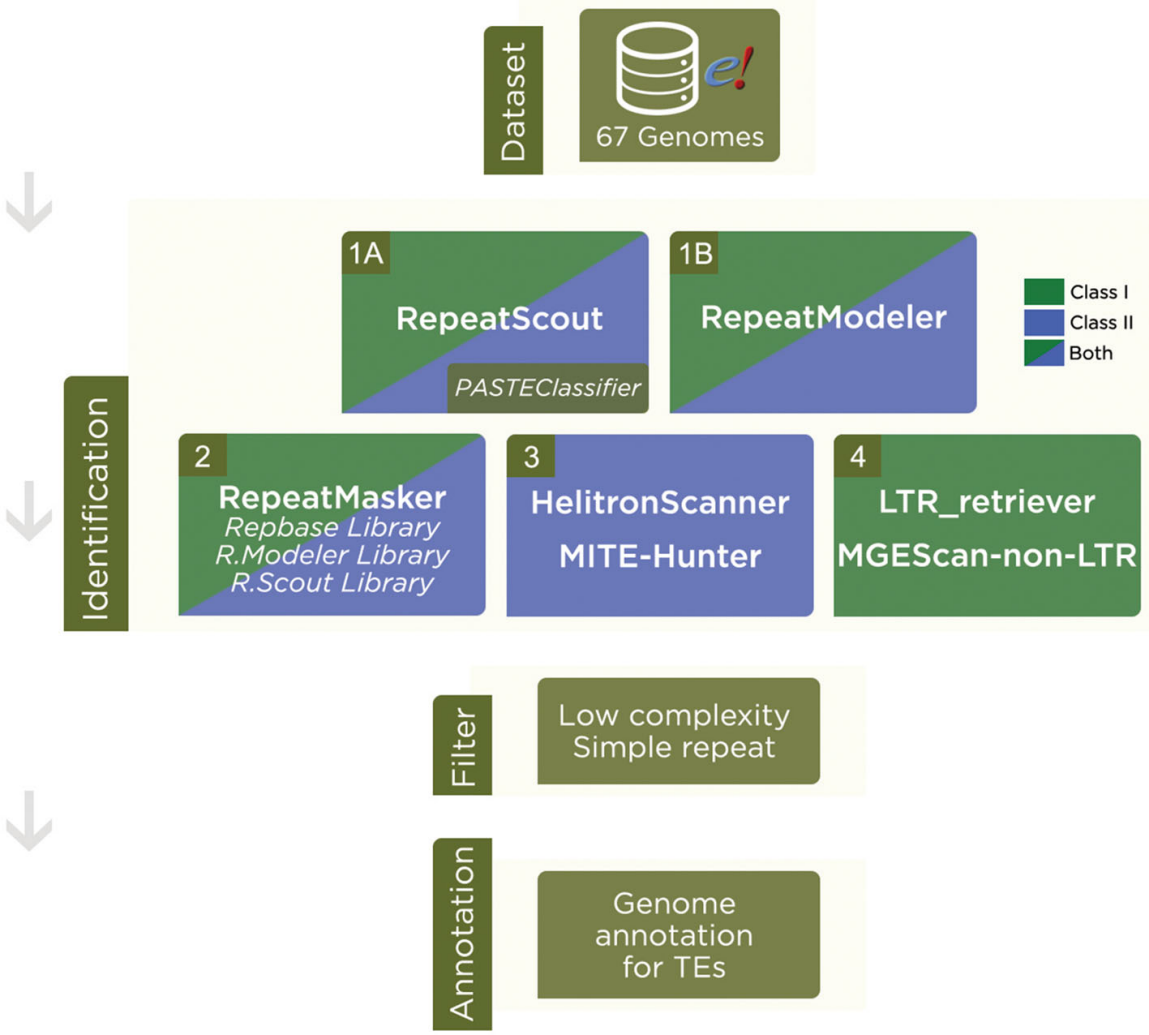
Steps in transposable elements identification. Dataset: Genome assemblies were downloaded from Ensembl Plants. Identification: 1A) RepeatScout was used to search for putative repetitive sequences and further classification by PASTEClassifier, resulting in a library. 1B) RepeatModeler was also used to find a consensus of TEs sequences. 2) RepeatMasker was run with Repbase library and libraries from RepeatModeler and RepeatScout. 3) For Class II - Subclass 2 TEs, we also used HelitronScanner and MITE-Hunter. 4) In order to find LTR and Non-LTR retrotransposons, we used LTR_retriever and MGEScan-non-LTR, respectively. Filter: A cut-off filter was applied to remove low complexities, simple repeats and other nomenclatures that were not classified into TEs. Annotation: In result of the pipeline, we have a Transposable Element annotation for each genome analyzed.

We used similarity-based methods and *de novo* techniques to build a collection of putative transposable elements, based on the SPTEdb pipeline. ^21^ We refined, extended and increased steps in order to produce a novel annotation ( Figure 1). Our reformulated steps (details in Supplementary Material 2, *Extended data*) guarantee a comprehensive knowledgebase of these TEs.

RepeatScout was performed separately; the output was unified in a library to be labeled by PASTEClassifier ^32^ and later combined into a final annotation. To automate the pipeline, an in-house framework in Perl language was developed for each software output to be uniformized, described in steps 1 to 4. A main script in Bash starts the process of automatization using Perl scripts. All steps were supervised by researchers, carefully checked, and the output was manually verified at each step for each genome. Records classified as low complexity, simple repeat and other nomenclature not related to Class I or Class II TEs were discarded.

Due to the extensive genome sizes of *Triticum aestivum* (14,5 Gb), *Triticum dicoccum* (10,4 Gb) and *Triticum turgidum* (10,4 Gb), we adapted our pipeline for their analysis, based on the approach of Jamilloux *et al.* ^24^ For these species, we applied our pipeline on chromosome 1 (which is the longest pseudomolecule), as the large genomes were eventually duplicated into new copies, increasing the number of these same repeats in the genome, and did not significantly impact discoveries related to new or different TEs families. ^24^

**TE evidence score**

**Figure 2.**
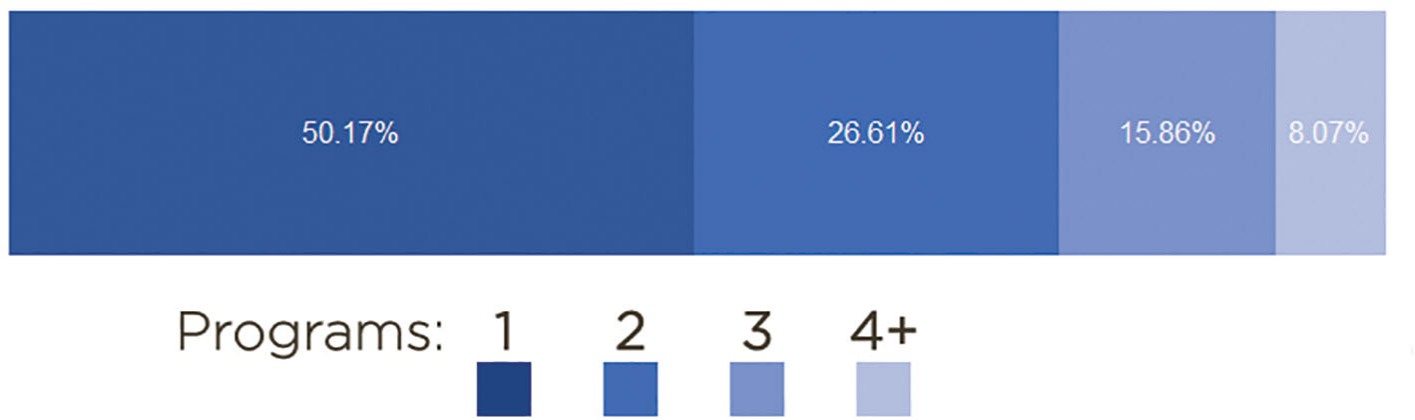
The TE Score: the average amount of sequence identification made by programs in all genomes.

To test the reliability of our TE annotation pipeline, we scored sequences that had duplicated annotation in the same loci ( Figure 2). We developed a statistical metric (labeled as TE-Score, shown in each record as the ninth column for each genome annotation file) that identify and ponder sequences types that have been identified by the programs. The TE-Score is a metric (0 to 1) that is given by

$$\mathrm{TE}-\text{Score}=\frac{\mathrm{QIP}}{\mathrm{QP}}$$

where QIP = Quantity of identification by program and QP = Quantity of programs. To illustrate an average of the amount sequences annotation by programs, see Figure 2.

### Correlation analysis

To test for correlations between genome size and transposable elements percentage by genome in base pairs, we first normalized using log10, and then we applied the Pearson Correlation Coefficient in SPSS version 25.

### Web implementation

APTE is hosted at the Universidade Tecnológica Federal do Paraná (Cornélio Procópio, PR, Brazil). It uses Debian 11 as operating system, Apache 2 as web server, PHP 5.6 as web programming language. We also used Zend Framework 2, which implements model, view, controller (MVC), a methodology for web development that can be expanded for any future additional functionality. On the front-end, we used HyperText Markup Language 5 (HTML5), Cascading Style Sheet 3 (CSS3) and JavaScript to perform dynamic functions that provide user-friendly navigation. A built-in genome browser (JBrowse, version 1.14.1) is available to visualize and download the data as well.

### Computational resources

To run the pipeline described in Figure 1, we used three platforms: (i) to runRepeatModeler, ^25^^,^^26^ RepeatScout, ^26^^,^^27^ RepeatMasker, ^27^ LTR_retriever, ^28^ MITE-Hunter ^29^ and HelitronScanner ^30^; (ii) to perform MGEScan-non-LTR ^31^ and PASTEClassifier ^32^; and (iii) to unify and filter outputs to the main annotation. The hardware utilized were (i) Xeon E7540 2.00 GHz 256GB memory, Xeon E5-2620v3 2.40 GHz 64GB memory, 2x Intel i7-3820 3.60Ghz 32GB memory and Intel i7-3820 3.60Ghz 64GB memory, (ii) Intel i7-3820 3.60Ghz 64GB memory, and (iii) Intel Core 2 Duo 2.4 GHz 8GB memory, a total of 30 physical cores and 456 GB of memory. In order to present a scale of time elapsed to measure, filter and standardize the results, we estimate that for the *A. thaliana* genome, the time needed to get the final annotation was ~18 hours, using all resources mentioned, including post-processing scripts (detailed on our website).

## Results and discussion

### Overview of TE portion

**Figure 3.**
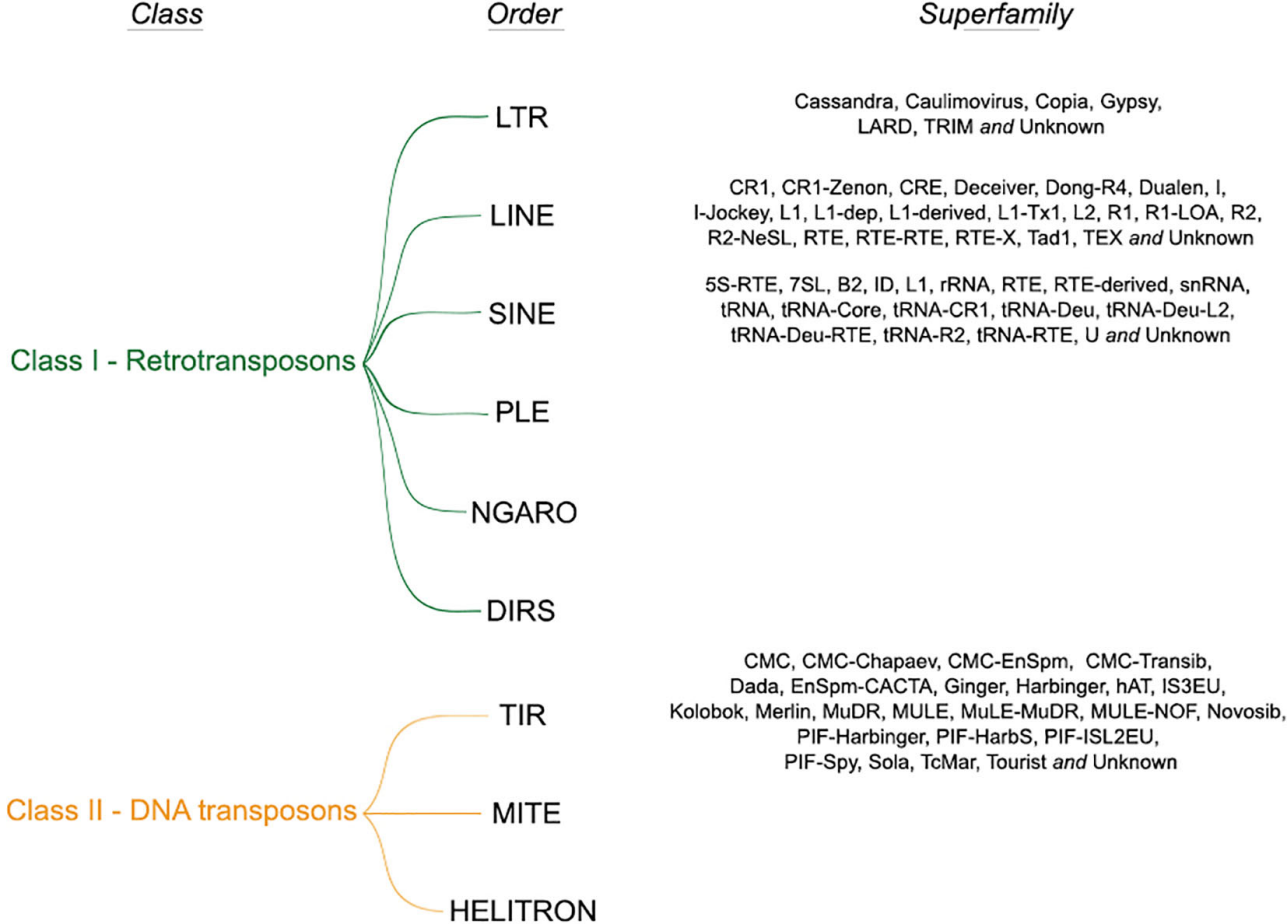
Class, order and superfamilies identified among the 67 plant genomes used in this study.

**Figure 4.**
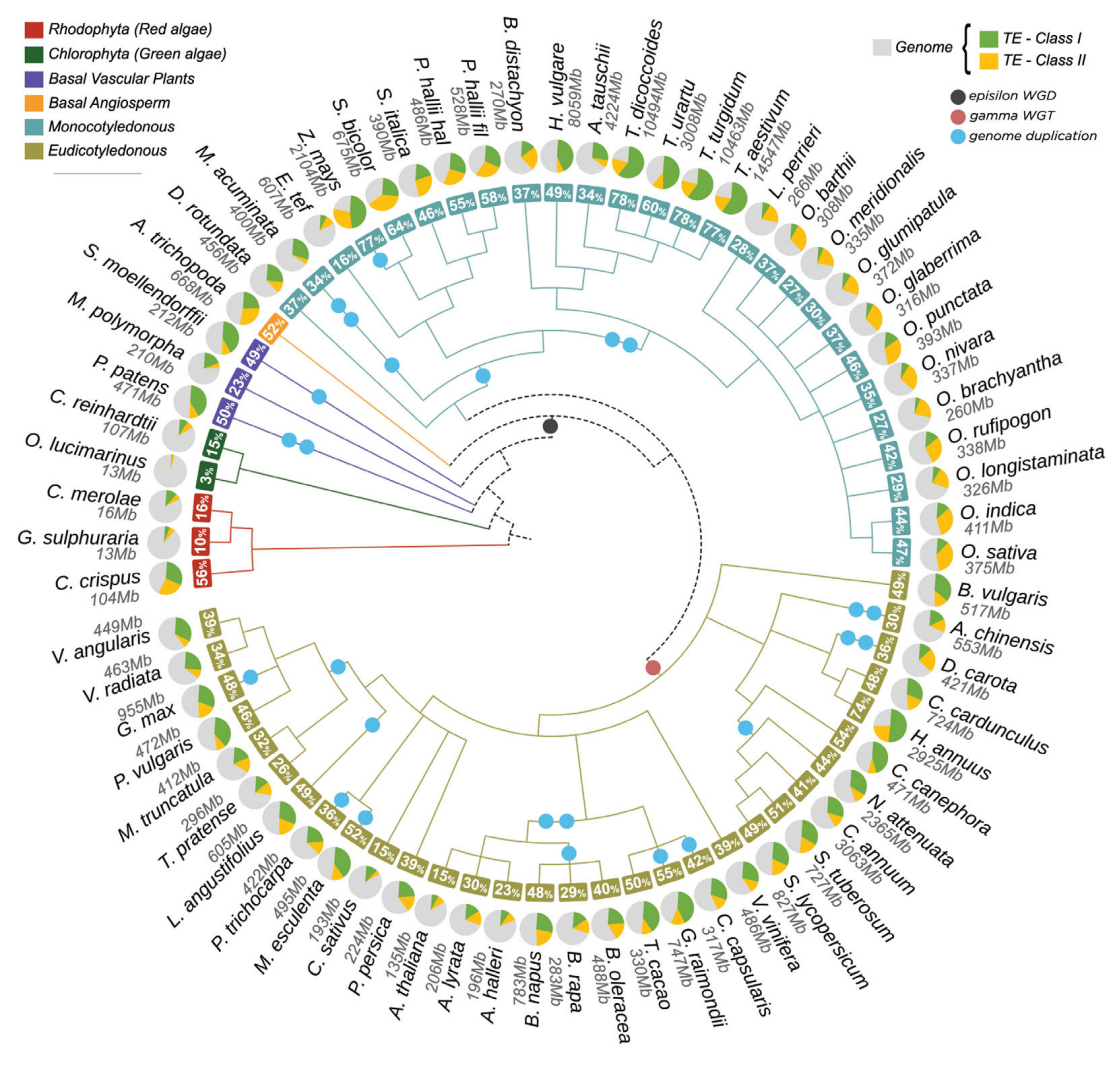
Overview of Class I and Class II composition of TEs in each genome organized in a phylogenetic tree.

We retrieved a total of 49,802,023 TE records from 67 plant genomes, representing a total of 47,992,091,043 (~47,62%) base pairs (bp) of the total genomic space. This information is distributed in ~57,36% (28,565,034) TEs organized into class, order and superfamily. In addition, ~42,64% (21,236,989) elements could not be assigned to any type of known TE and they were labelled as unknown. They likely represent chimeric and/or partial elements for which we were not able to perform the full classification. For known TEs, we identified that ~62,85% were retrotransposons, and ~37,15% were DNA transposons. All assigned classifications of TEs identified along the 67 genomes are shown in Figure 3. The distribution of TEs in the analyzed genomes are somewhat similar ( Figure 4), especially in genomes that have a shorter phylogenetic distance (e.g., *Oryza* spp, *Triticum* spp). However, even close-related genomes exhibit uneven TEs distribution (e.g., *Arabidopsis* spp). Two main hypotheses might explain the variation of TE content: (a) different evolutionary stories, since the two major genome duplication events are shared by all seed plants ( *epsilon*) and flowering plants ( *gamma*), followed by the lineage-specific duplication events, ^20^ and (b) specific pressures to maintain, expand and purge TEs in each lineage.

**Figure 5.**
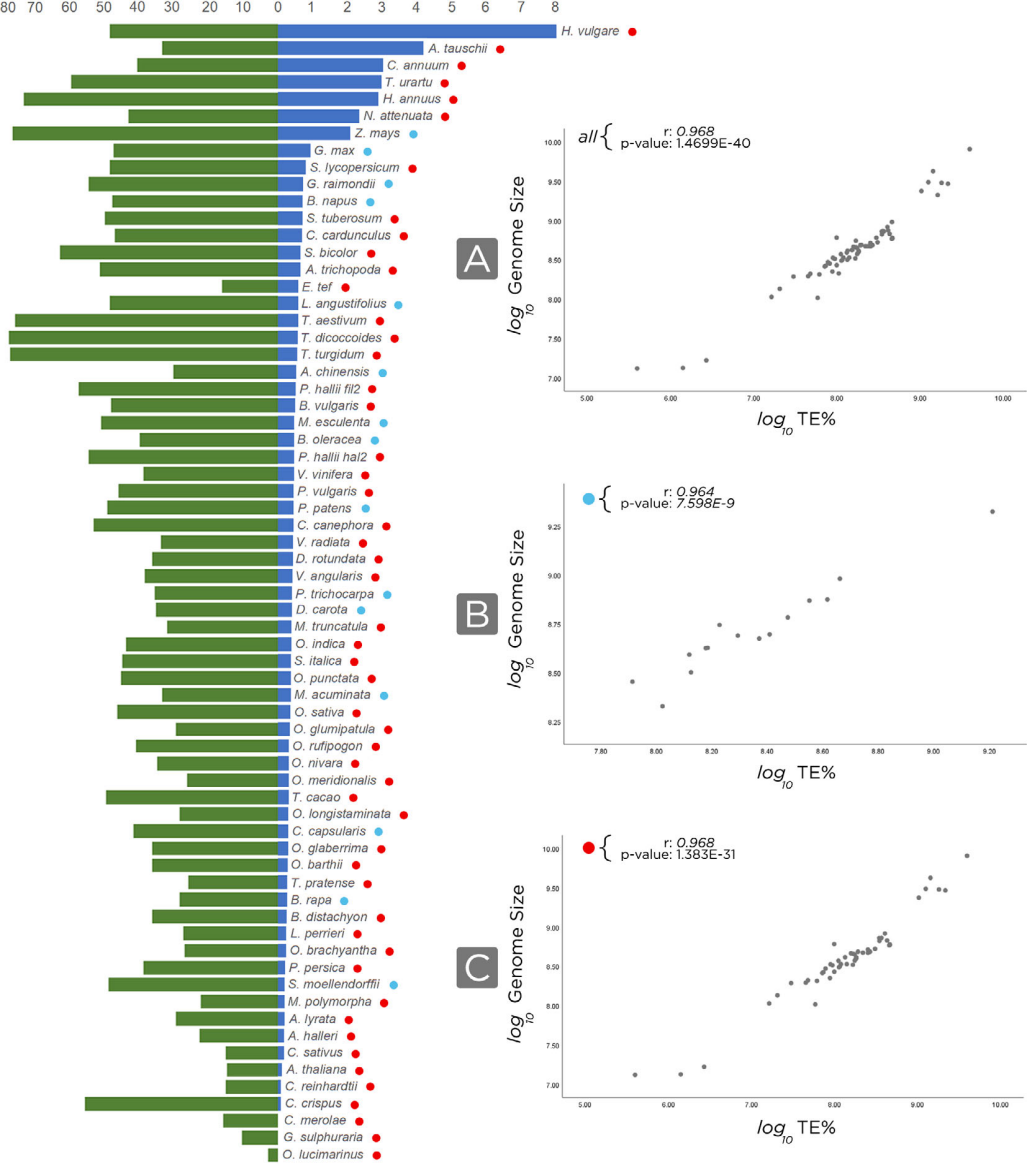
Correlation between genome size and TE content. On the left, the bar chart in blue, the genome size (in Gb), and, in green, the transposable elements distribution in analyzed genomes (in percentage). On the right, we normalized, in base pair, genome size and TE using log(10) and then we correlated (Pearson) the genome size by transposable elements. *r* and *p-value* are shown in the top-left of each chart. A) Using all the 67 annotated genomes; B) For all genomes with recent WGD (Whole Genome Duplication) events, blue circles; C) Excluding genomes that experienced recent WGD, red circles.

We have noted that our approach permitted better TE annotations in genomes assembled at chromosome scale, and we also observed that the amount of TEs is generally related to the genome size, since larger genomes have higher occurrences of TEs ( Figure 5). However, for incompletely and draft-assembled genomes, it tends to decrease the number of TEs, once the assembly into small parts (scaffolds or contigs) may impact the genome assembly quality, collapsing repeated contigs (mostly TE- derived) and interfering with the proper identification of these TEs.

### TE database comparison

To compare the results of the identification performed and to ensure the reliability (details in Supplementary Material 1, *Extended data*) of our approach, we used SPTEdb ^21^ annotation data of the genome *Populus trichocarpa* (black cottonwood), which is explored in Table 1. The second comparison of TE annotations was performed for the *Glycine max* (soybean) genome, in which we used SoyTEdb ^22^ to compare the amount vs. type of TEs, shown in Table 1. A third comparison used data from GrTEdb ^23^ to explore the amount of TEs in *Gossypium raimondii* (cotton), available in Table 1.

**Table 1.** Transposable elements amount annotated in *Populus trichopoda, Glycine max* and *Gossypium raimondii* by class, order and superfamily compared to other databases which have their own annotation.

			*Populus trichopoda*		*Glycine max*		*Gossypium raimondii*	
			Our work	SPTEdb	Our work	SoyTEdb	Our work	GrTEdb
Class	Order	Superfamily	Quantity					
Class I	LTR	Cassandra	17	-	1,126	-	157	-
		Caulimovirus	469	6	574	-	1,479	-
		Copia	28,259	1,557	60,399	13,318	50,543	2,929
		Gypsy	57,505	5,587	92,396	19,052	161,281	10,368
		LARD	142	-	6,804	-	2,832	-
		TRIM	1,164	-	3,698	-	2,239	-
		Pao	-	140	-	-	-	-
		Unknown	38,146	-	22,978	-	42,814	-
	LINE	CR1	84	-	-	-	-	-
		CRE	-	-	1	-	-	-
		I	173	-	168	-	-	-
		L1	3,472	87	11,897	-	13,018	299
		L2	695	-	177	-	215	-
		RTE	362	-	3,927	-	78	-
		TEX	2	-	2	-	1	-
		Unknown	4,484	-	6,119	182	3,572	-
		rRNA	174	-	193	-	509	-
		tRNA	18,775	-	2,784		850	-
		snRNA	-	-	64	-	-	-
		Unknown	12,922	-	4,152	-	4,485	-
	NGARO	-	-	6	-	-	-	-
	PLE	Penelope	-	-	14	-	4	-
	DIRS	-	3	5	2	-	1	-
Class II	TIR	CMC-EnSpm	7,031	3	26,395	-	1,547	-
		Crypton	-	-	535	-	-	-
		Dada	-	-	-	-	58	-
		EnSpm-CACTA	3,590	-	4,596	65	1,419	275
		Harbinger	584	-	1,182	-	1,036	-
		hAT	3,383	17	3,348	65	4,837	-
		hAT-Ac	3,301	-	9,676	-	22,333	-
		hAT-Charlie	-	-	180	-	134	-
		hAT-Tag1	11,022	-	4,612	-	4,650	-
		hAT-Tip100	935	-	2,805	-	7,733	-
		Maverick	-	-	-	-	115	-
		MuDR	2,246	-	15,920	2,373	9,933	12
		MuLE-MuDR	2,712	6	27,566	-	11,207	-
		Novosib	74	-	577	-	55	-
		PIF-Harbinger	6,430	3	8,157	90	4,342	435
		Pong	-	-	-	12	-	-
		Sola1	5	-	128	-	61	-
		Tc1-Mariner	623	-	472	9	868	
		TcMar-Pogo	333	-	78	-	-	-
		TcMar-Stowaway	-	-	2,920	-	-	-
		Unknown	5,379	2,770	5,664	-	7,451	-
	MITE	-	1,426	78	9,355	3,333	11,661	-
	Helitron	-	51,532	1,340	5,860	82	5,307	14
Unknown	-	-	232,104	-	619,524	-	618,413	-

## Conclusion

Our analysis brought an exhaustive, systematic and comprehensive genome identification in plant genomes, using seven programs to annotate TEs in plant genomes. In both TE classes, several orders and superfamilies were found ubiquitously in all genomes. Additionally, 21,236,989 out of 49,802,023 mapped TE sequences could not be classified into any of the nomenclatures known for TEs, and were labeled as “Unknown” in GFF3, a standard file format for gene annotation.

For plant species whose TE complement may be quite well-annotated, i.e., *Arabidopsis thaliana*, we yielded an increased number of identified TEs. In species with less curated annotation in Ensembl, we were able to deliver a more detailed identification of TEs. For example, in three particular genomes, i.e., *Populus trichocarpa* (black cottonwood), *Glycine max* (soybean) and *Gossypium raimondii* (cotton), we increased the TE identification levels by 2,295%, 900% and 2,643%, respectively. We observe that for several other genomes, new types of TEs were identified and annotated; this ensures that our pipeline delivers not only the same TE identification, but also new ones, making the annotation process possible to use for any species.

In this study, we contributed to expand the knowledge on TEs, by providing a large-scale, organized and standardized TE Atlas. We integrated all annotations to make it available to download in each genome separately from the Atlas of Plant Transposable Elements (APTE) website. An example how our pipeline works using the *A. thaliana* genome, software dependencies, and in-house scripts developed, which can be downloaded, used and changed freely, are available from https:// github.com/alerpaschoal/apte_pipeline/.

## Data availability

### Underlying data

All data underlying the Plant TE Atlas is available in the portal http://apte.cp.utfpr.edu.br/.

### Extended data

Zenodo: Datasets from An Atlas of Plant Transposable Elements, https://doi.org/10.5281/zenodo.5672122. ^33^ 

This project contains the following extended data:

- SuppMat_1.xlsx (the gen ome assembly reference access from Ensembl Plants species used)
- SuppMat_2.docx (a brief transposable elements annotation steps used in this work)

Data are available under the terms of the Creative Commons Zero “No rights reserved” data waiver (CC0 1.0 Public domain dedication).

Analysis code available at: https://github.com/alerpaschoal/apte_pipeline/

Archived code at time of publication: https://doi.org/10.5281/zenodo.5672122

License: CC0
