# Supplementary material for: An Atlas of Plant Transposable Elements
Source: F1000Res. 2021 Nov 24;10:1194. [Version 1] doi: 10.12688/f1000research.74524.1 (PMC8729191; doi:10.12688/f1000research.74524.1)
Supplement: Peer review history

# Peer review: Reviewer response for version 1

**DOI:** 10.5256/f1000research.78290.r116755

**Published:** 2022-01-04

**Authors:** Adriana Ludwig

**Affiliations:** 1 Laboratory of Applied Health Sciences and Technologies (LaCTAS), Carlos Chagas Institute, Oswaldo Cruz Foundation (Fiocruz), Curitiba, Brazil

Pedro *et al.,* presented an atlas of transposable elements from 67 well-assembled plant genomes. The full TE annotation is provided in the APTE database that is informative and straightforward. It is a very good source of data for other researchers that might be interested in proceeding deepest analysis of a specific group of elements. The authors have identified a great number of repetitive sequences in each genome. My biggest concern is related to the sequences that fail to be classified. These “Unknown” sequences make up a huge proportion of all the repetitive sequences found. Even with the filtering of low complexity and simple repeat sequences, they could still be false positive. The authors could mention this possibility. Moreover, the difference in the amount of TE identification between the two databases shown in Table 1 is impressive. My doubt is whether there is a possibility that part of this difference is caused by the artificial "breaks" of TE copies into a greater number of fragments. Finally, it is not clear if the authors have filtered short sequences that could also be false positive.

**Minor suggestions/comments:**

- Page 6: “evolutionary stories”. I believe “evolutionary histories” would be more appropriate.
- Concerning the TE classification. In Figure 3, I suggest some modifications to become more accurate regarding the TE classification (according to Wicker *et al*.;2007 ^1^):

1. DIRS and Ngaro are mentioned as orders. The correct is to include both as superfamilies of order DIRS.
2. MITE is not an order. They are non-autonomous elements from order TIR. In the same way, LARD and TRIM are not superfamilies, but non-autonomous elements related to some LTR superfamily. I suggest a change to “Superfamily/Group” since some groups specified are not independent established superfamilies, but rather groups/clades of a superfamily. The same occurs in Table 1. Moreover, in Table 1, Crypton should be placed as an order.

Is the work clearly and accurately presented and does it cite the current literature?

Yes

If applicable, is the statistical analysis and its interpretation appropriate?

Yes

Are all the source data underlying the results available to ensure full reproducibility?

Yes

Is the study design appropriate and is the work technically sound?

Yes

Are the conclusions drawn adequately supported by the results?

Yes

Are sufficient details of methods and analysis provided to allow replication by others?

Yes

Reviewer Expertise:

Transposable elements

I confirm that I have read this submission and believe that I have an appropriate level of expertise to confirm that it is of an acceptable scientific standard.

## References

[1] : A unified classification system for eukaryotic transposable elements. *Nature Reviews Genetics* .2007;8(12) : 10.1038/nrg2165 973-982 10.1038/nrg2165 17984973

# Peer review: Reviewer response for version 1

**DOI:** 10.5256/f1000research.78290.r101071

**Published:** 2021-12-01

**Authors:** Gökhan Karakülah

**Affiliations:** 1 Izmir Biomedicine and Genome Center, Dokuz Eylül University, Izmir, Turkey

In this work, Pedro and colleagues present a novel transposable elements database for plants using publicly available genome sequences from the Ensembl Plant database. The methodology was clearly described and seems reproducible. The main output of the current work is a publicly available database named APTE and its interfaces are elegantly designed. All transposon annotations are available without any restriction and the database is easy-to-use.

 I believe this unique and valuable database has potential to help researchers in both computational and wet lab analyses of mobile elements.

Is the work clearly and accurately presented and does it cite the current literature?

Yes

If applicable, is the statistical analysis and its interpretation appropriate?

Not applicable

Are all the source data underlying the results available to ensure full reproducibility?

Yes

Is the study design appropriate and is the work technically sound?

Yes

Are the conclusions drawn adequately supported by the results?

Yes

Are sufficient details of methods and analysis provided to allow replication by others?

Yes

Reviewer Expertise:

Bioinformatics, Genomics, Mobile elements, Repetitive DNA, Non-coding RNAs

I confirm that I have read this submission and believe that I have an appropriate level of expertise to confirm that it is of an acceptable scientific standard.
